# Dangerous Newspapers Receipts

**Published:** 1855-04

**Authors:** 


### DANGEROUS NEWSPAPERS RECEIPTS.

No man who values his own health and life, and those of his family, should pay any attention to any newspaper recipe of a medicinal character. The door should be shut against all family newspapers which habitually insert such things, because mainly there are various medicines which do a striking good when taken once or twice, but which cause poisonous effects if taken four or five times or days in succession. A dose of calomel for example, will in many instances, when judiciously and appropriately given, cause prompt and permanent changes in the system of the most gratifying character, but very often, if a dose is taken three or four days in succession, even if "worked off," salivations of the most dreadful character are the result.

Then, there are other medicines which produce apparently good results, but when they are discontinued, the person inevitably dies. Arsenic is reported to be of this character, that if carriage horses are allowed a small portion daily, they soon become fat, the hair becomes sleek and shiny, and they foam at the mouth profusely when put in harness; the same authority asserts, that in Austria and Hungary, the young use it to give color to the cheek and fulness of flesh, thus making them more attractive to the opposite sex, but if discontinued, especially if suddenly, they soon begin to pine away and die. We have a very familiar, and most unfortunately a very frequent illustration of this fact in the use of brandy or porter, and other liquors, they at first exhilarate both body and mind, and seem to place the drinker in excellent health, but when the habit has been long established, it is almost death to abandon it, the want of it is at times so resistless, that a recent convict, after exhausting all his ingenuity to get a drink of his accustomed "BEVERAGE!" seized an axe in the penitentiary, and in an instant cut off his hand as if by accident, called at once for a bowl of brandy, as if to staunch the blood; the keepers were thrown off their guard, in a moment he thrust in the bleeding stump, the next, the blood and brandy had passed down his throat.

Let the reader then keep in view the apparently good effects of some remedies for the present, with their subsequently destructive agencies, and make a practical use of it ever hereafter, in reading newspaper recipes.

Here is one, for example, which has been published and republished all over the country.

FOR MAKING (GOOD?) CORN BREAD.—One pound of Indian, that is, corn-meal, pint and a half of milk, five eggs, a piece of butter as large as a hen's egg, soda as large as a large pea, and a level teaspoon of cream of tartar; bake it three quarters of an hour.

So much for soda and cream of tartar in our bread. Another editor recommends as an excellent beverage:

DANDELION COFFEE.—Made by washing the roots of the common field dandelion clean, without scarifying them; cut them up as fine as coffee berries, roast, grind, and prepare in the same way.

Another recommends

Columbo water, as a safe stimulant for languid appetite. Take four drachms of the bruised columbo root, one drachm of bitter orange peel, and two drachms of fresh liquorice root; add a quart of soft water, and simmer as gently as possible over a slow fire until half the bulk of the water is evaporated; then strain the liquor, filter it, add one-sixth of good pale brandy, bottle it, and take an hour before dinner, of this mixture, a third of a wine-glass, filling up the glass with cold water.

And as if not satisfied with poisoning our bread, giving us physic for coffee, and then drenching our stomach with medicated water; another still advises how to kill our cooks and washerwomen, by urging them

To save Labor in Washing,—By putting a tablespoon or two of spirits of turpentine in the water; and thus with the aid of villanous camphene for lamps, being essentially turpentine itself, we are likely to be poisoned, blown up, burnt up and teetotally killed, master, mistress, children, servants, dogs, cats, and all. Surely we are getting to a pretty pass; presently we will not be able to turn round without taking a dose of physic; and as if to make certainty more sure, lest the cook should fail to put the soda or saleratus or cream of tartar, it is put in by the manufacturer ready to hand, in the shape of "Patent flour." And in case we should not prefer the dandelion coffee, and desire the pure coffee, old Java, our grocer, for our own good—or his—intersperses it with other ingredients, for a case came up for trial a few days since in New York, wherein one party sued another for the value of forty bags of peas. The plaintiff was a coffee roaster, and had contracted with the defendant for 250 bags of peas, which, it appeared, were to be ground up with the coffee.—Some curious developments came out in the course of the trial, showing the extent to which peas, chicory and other substances are used for the article which is sold as *pure ground coffee*.

People seem to think that because an article is familiar to them from every-day use, there can be no great harm in it, and forthwith baptize it as *simple.* Well, look at the effect of one of the most familiar of these *simples.*

Essence of Peppermint.—A little of it in candy for our children on holidays may not be injurious, nor will a little peppermint tea, or a drop or two now and then in a little water for babies. On some occasions it may be allowable.

But a case is alluded to recently near Killingly, Connecticut, where a man who had been a moderate drinker of spirituous liquors and finding his supplies cut off, resorted to peppermint water, which in a short time killed him. And soda is another simple, made as it is out of the ashes of sea weed, as saleratus is made of the ashes of wood, so simple that our wives drink it down every day, and look upon it with admiration, as a prompt antidote for sour stomach, that is, an over hearty dinner, and finding it so prompt and efficient, and having a strong appetite for the very next meal, they over-eat again, and again resort to the soda bottle; but in a few days the dose must be increased or it does no good, and before one is aware of it, there is a necessity for its being taken as regularly as the daily meals; next, there must be a steady increase in amount, or the most intense suffering is the result. Not long ago, as appears from the report of a coroner's inquest in London, a gentleman was standing at the door of his daughter, whom he had called to see a moment, and while talking to her dropped down dead. On examining his body after death, it was found to be the result of an impacted mass of solid soda, which had accumulated in the tract of the bowels, he having resorted to it daily to remove flatulence and "sour stomach."

Turpentine is another simple, just as simple as the people are who persist in its use for burning in their lamps, notwithstanding a day scarcely passes in which is not chronicled the death of some careless servant, or child, or parent—the terrible death of burning.

A person may well wash out some clothing a single time without much trouble and without any bodily injury, but suppose it is repeated every week or oftener, we cannot otherwise than expect to witness the legitimate results of its over application, such as violent inflammation of the skin, with extensive eruptions from all parts of the body. If applied largely to the breast of a horse it will produce death in a few hours; and yet some thoughtless editor has believed himself to do a public service by recommending to washerwomen as a labor-saving agent the common use of spirits of turpentine.

The best corn bread in the world is made by the negroes in the west and south-west with the meal, a little salt, a lump of hog's lard or butter, and as much water or milk as will give it a proper consistence, and put in immediately to bake without any other ingredient whatever.

As for washing clothes, a good soak over night in soft or rain water, common soap, and a pair of willing hands, these are all that is necessary to make the cleanest "linen" in the world and the sweetest; for Beau Brummel declared he knew no perfume equal to that of a well-washed garment.

Therefore, reader, abnegate, abominate and exterminate all newspaper "*receipts*" whatsoever, unless such papers are of a scientific character, such as the "*Scientific American.*"

